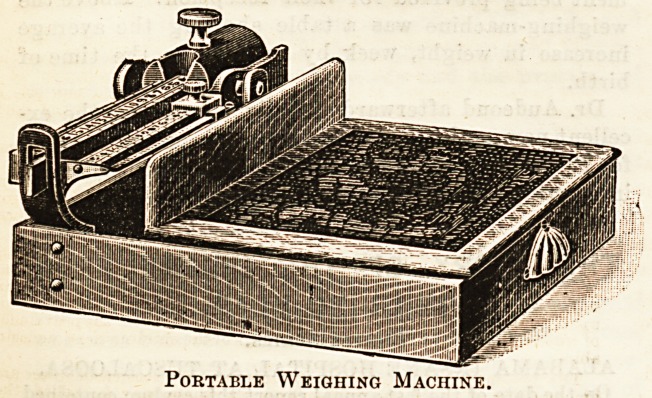# Practical Departments

**Published:** 1895-09-07

**Authors:** 


### PRACTICAL DEPARTMENTS.

Messrs. Thornhill and Co., whose delightful shop in Bond Street is a joy to those who know it, have brought out a most useful contrivance in the matter of weighing machines. The

Portable Weighing Machine.

"Miniature Portable Personal Weighing Machine," the "Cholmondeley" (after its inventor the Marquis of that name), to give it its full title and dignity, is a compact little affair, small enough to be carried without difficulty, and registering with accuracy by ounces up to twenty-five stone by the arrangement shown in our illustration (which we give by permission of Messrs. Thornhill), no weight being required. The price, in polished oak, with carpet covered platform, is £6 15s., but we believe Messrs. Thornhill are in the habit of making a reduction in the case of nursing homes and charitable institutions. There is a considerable demand, as may readily be imagined, in this direction for so convenient a necessary as a weighing machine must be considered from a medical point of view. For doctors, too, this should prove a great boon, and we shall expect to hear that it is being largely adopted by the profession. The ordinary apparatus for registering weight is of necessity more or less clumsy, and Messrs. Thornhills' novelty may therefore be said with truth to "supply a real want."

## Figures and Tables

**Figure f1:**